# Comparative effects of MAD and CPAP on heart rate in obstructive sleep apnea

**DOI:** 10.1007/s11325-025-03494-x

**Published:** 2025-10-06

**Authors:** Pasquale Tondo, Mauro Lorusso, Fariba Esperouz, Giuseppina Giannini, Giuseppe Burlon, Matteo Pio Natale, Roberto Sabato, Giulia Scioscia, Monteleone Leonardo, Giulio Trono, Michele Tepedino, Lucio Lo Russo, Donato Lacedonia, Domenico Ciavarella

**Affiliations:** 1 Department of Medical and Surgical sciences, University of Foggia, Foggia, Italy; 2 Institute of Respiratory Diseases, Policlinico Riuniti of Foggia, Foggia, 71122 Italy; 3 Department of Clinical and Experimental Medicine, Dental School of Foggia, University of Foggia, Foggia, Italy; 4 Department of Biotechnological and Applied Clinical Sciences, Dental School of L’Aquila, University of L’Aquila, L’Aquila, Italy; 5 Respiratory Medicine Unit, Universo Salute Opera Don Uva, Via Rovelli, 50, Foggia, 71122 Italy

**Keywords:** Obstructive sleep apnea, Polysomnography, MAD, CPAP

## Abstract

**Purpose:**

This observational study aimed to compare the effectiveness of mandibular advancement devices (MAD) and continuous positive airway pressure (CPAP) therapy in patients diagnosed with obstructive sleep apnea (OSA), with a specific focus on changes in heart rate parameters.

**Methods:**

Fifty-two patients treated with MAD were compared with fifty-two patients treated with CPAP. All participants underwent sleep study prior to the initiation of therapy (T0), followed by a control sleep study after a (3-month) predefined treatment period (T1). Changes in cardiac parameters between baseline (T0) and post-treatment (T1) were analyzed both within each group (MAD and CPAP) and between groups. To enhance comparability between patients treated with MAD and those treated with CPAP, a propensity score matching approach was applied based on pre-treatment variables, in order to minimize selection bias and optimize the validity of the between-group comparison.

**Results:**

For intragroup comparisons, both the MAD and C-PAP groups demonstrated statistically significant reductions in AHI and ODI following treatment (*p* < 0.001). Additionally, a modest but significant decrease in mean heart rate was noted in both groups (MAD: *p* = 0.033; CPAP: *p* = 0.002). For intergroup comparisons, both treatment modalities resulted in a significant reduction in AHI and ODI values. Regarding heart rate parameters, no statistically significant differences were observed between the two groups after treatment for mean HR (*p* = 0.350), minimum HR (*p* = 0.602), or maximum HR (*p* = 0.942).

**Conclusion:**

The use of MAD and CPAP leads to significant improvements in heart rate in patients with OSA. However, no significant differences were found between the two treatment modalities in terms of heart rate reduction.

**Supplementary Information:**

The online version contains supplementary material available at 10.1007/s11325-025-03494-x.

## Introduction

 Obstructive sleep apnea (OSA) is a common sleep disorder [1] characterized by repeated airway collapse during sleep, leading to intermittent hypoxia and fragmented sleep [2, 3]. These pathophysiological events are strongly associated with increased cardiovascular and metabolic risk [4, 5]. OSA is recognized as an independent risk factor for various cardiovascular conditions, including hypertension, heart failure, coronary artery disease, and both cardiovascular and cerebrovascular disorders [6–8]. The link between OSA and cardiovascular diseases is mainly driven by factors such as sympathetic nervous system activation, oxidative stress, and widespread systemic inflammation [9, 10]. Additionally, it has been observed that OSA can exacerbate cardiovascular diseases, which, in turn, may worsen the OSAS condition, leading to a harmful feedback loop. Consequently, early identification and treatment of OSAS may play a crucial role in alleviating or improving the impact of cardiovascular diseases [11]. OSA profoundly affects heart rate (HR) regulation due to recurrent episodes of apnea and hypopnea causing intermittent hypoxia and autonomic nervous system imbalance. These events trigger cyclical HR fluctuations, typically characterized by bradycardia during apnea and tachycardia upon arousal, which increase cardiovascular risk. Studies have demonstrated that patients with OSA exhibit elevated resting and nocturnal HR compared to healthy individuals, reflecting sustained sympathetic activation [12]. In fact, numerous studies have shown that alterations in the circadian rhythm of heart rate in individuals with OSA may contribute to the development of cardiometabolic diseases [13].

Effective treatment of OSA contributes to improved cardiovascular health by stabilizing heart rate regulation and lowering the risk of cardiovascular complications associated with the disorder. Early identification and effective treatment of OSA are therefore critical not only to improve patients’ quality of life but also to prevent the progression of potentially life-threatening cardiovascular and metabolic complications.

Continuous positive airway pressure (CPAP) therapy is the standard treatment for OSA which helps maintain of airway patency during sleep [14]. Despite its effectiveness, patient adherence to CPAP therapy tends to be low (Pepin), leading to growing interest in alternative treatment options. The American Thoracic Society guidelines suggest that individuals with OSAS should engage in a holistic lifestyle intervention program, which includes regular physical activity and weight loss [15]. A viable alternative to CPAP for treating mild to moderate OSA is the use of a Mandibular Advancement Device (MAD). Recent evidence suggests that MADs may offer cardiovascular benefits comparable to CPAP [16], particularly in terms of reducing oxidative stress, inflammation, and improving endothelial function, likely due to greater patient adherence [17, 18]. These findings support MADs as an effective option, especially in patients with mild-to-moderate OSA or those intolerant to CPAP [19].

Despite the growing body of evidence supporting the cardiovascular benefits of both CPAP and MAD therapy, few studies have directly compared their impact on heart rate dynamics and event reduction in real-life clinical settings. To date, the effects of CPAP and MAD on heart rate remain unclear, as no studies have directly compared these therapeutic devices. This gap is particularly important given the high prevalence of cardiovascular comorbidities in patients with OSA. Addressing it may improve understanding of treatment effects and support clinicians in developing more effective and individualized therapeutic strategies.

The present study aims to evaluate and compare the effects of CPAP and MAD on night-time heart rate fluctuations (minimum, mean, and maximum HR) and on the reduction of respiratory events, in a real-world population of patients with OSA. By applying a propensity score matching approach, we sought to ensure comparable baseline characteristics between treatment groups and to provide a more accurate assessment of their respective physiological impacts under routine clinical practice conditions.

## Materials and methods

### Study design

This study was conducted in accordance with the Strengthening the Reporting of Observational Studies in Epidemiology (STROBE) guidelines for observational research [20]. Data were retrieved retrospectively and analyzed anonymously. All participants provided written informed consent prior to inclusion in the study. A total of 104 adult patients with a confirmed diagnosis of OSA were retrospectively included. Participants were enrolled according to the following inclusion criteria: age over 50 years, treatment with CPAP or MAD, diagnosis of moderate to severe obstructive sleep apnea syndrome (OSAS), and a body mass index (BMI) below 30 kg/m². Exclusion criteria included smoking habits, neurological disorders, cardiac conditions, and a history of stroke or heart failure. The cohort was split into two treatment arms: 52 patients treated with MAD, and 52 patients with CPAP. Patient data were collected from consecutive clinical records at the Department of Orthodontics and Respiratory Medicine Unit, University of Foggia, Italy, over the period March 2022 to October 2024.

Each individual underwent a diagnostic sleep study prior to therapy initiation and a follow-up polysomnographic evaluation after a predetermined treatment interval. The collected data included the polysomnographic indices: apnea-hypopnea index (AHI) and oxygen desaturation index (ODI), as well as mean, minimum, and maximum heart rate, measured for each patient before (T0) and after treatment (T1). These parameters were compared to assess the impact of two treatments on cardiac function (HR min, mean and max).

### MAD protocol

**Fig. 1:**
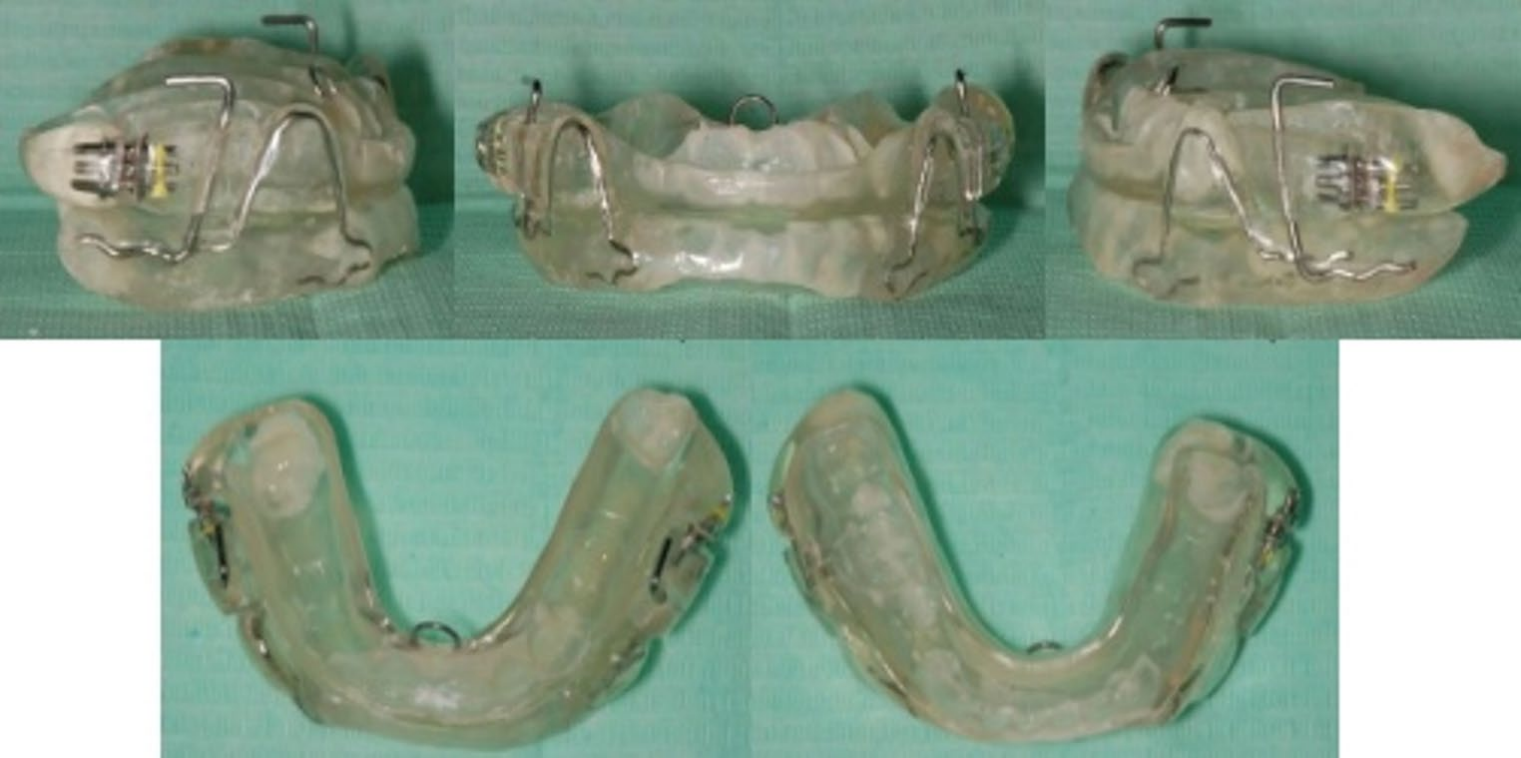
IMYS appliance

The appliance employed was the It Makes You Sleep (IMYS, Salcuni Dental Laboratory, Italy) device (Fig. 1), a custom-made and titratable MAD. The IMYS device consists of two resin splints connected by two vertical stainless-steel bars and three screws. The vertical bars are inserted into a vertically oriented slot, located mesially to the screws, and are embedded within the resin of both the upper and lower splints. These components allow for limited vertical and lateral mandibular movements while preventing full mouth opening. Additionally, the upper splint included a lingual loop to improve tongue posture [21] and stainless-steel wire stabilizing bars to reinforce the appliance during mandibular advancement. A functional mandibular evaluation was performed for each patient. Impressions of both the maxillary and mandibular arches were captured using the iTero intraoral scanner, generating digital models that were subsequently mounted on an articulator. Being titratable, the device was initially set to a mandibular advancement corresponding to 70% of the patient’s maximum protrusive range. Following two weeks of treatment, the mandibular position was modified in small increments (1–2 mm) to achieve the best possible therapeutic fit for each patient.

### CPAP protocol

Participants began CPAP titration following the diagnosis of OSA, in accordance with the clinical guidelines of the American Academy of Sleep Medicine [22].

Titration was conducted using an automatic positive airway pressure (APAP, ResMed Autoset Airsense 10, ResMed, Italy) device with the goal of identifying the optimal therapeutic pressure (fixed CPAP, ResMed Airsense 10, ResMed, Italy). The device used, equipped with advanced algorithms, could adjust the delivered pressure in real time, breath by breath, continuously monitoring respiratory parameters such as inspiratory flow, snoring level, and AHI index.

Titration was performed at home in cases where the time spent with oxygen saturation below 90% (T90) was less than 30% of the total recorded time. Conversely, when T90 > 30%, titration was carried out in a hospital-based pneumology unit.

After three consecutive nights in APAP mode, the fixed therapeutic pressure for CPAP was set. At this stage, the Expiratory Pressure Relief (EPR) function was activated, limited to the initial ramp phase, to improve tolerance and respiratory comfort. The selected EPR level corresponded to a reduction of 1 cmH₂O during exhalation. Following the final pressure setting, each participant continued treatment for two consecutive nights with fixed-pressure CPAP to assess the correction of respiratory events. Finally, all patients adapted to CPAP underwent a follow-up RPG to verify the effectiveness of the therapeutic setting achieved [22].

### Sleep study analysis

All participants underwent overnight diagnostic type 3 polysomnography/respiratory polygraphy (RPG, NOX T3, MedicAir, Italy) [23] at baseline (T0) and after 3 month of the assigned treatment (T1). The RPG included continuous monitoring of multiple physiological signals: airflow, thoraco-abdominal respiratory movements, oxygen saturation (SpO₂), heart rate and body position. The sleep recordings were manually scored by expert somnologists according to AASM criteria [24].

The following parameters were analyzed from the HST recordings for subsequent analysis for assessing the responses to treatment with MAD and C-PAP: AHI, ODI, and mean, minimum, and maximum heart rate (HRmean, HRmin, HRmax).

All patients included in the analyzed sample demonstrated proper adherence to the prescribed therapy. Both MAD- and CPAP-treated participants followed the therapeutic instructions, resulting in high compliance. No side effects were observed in the CPAP group. In the MAD group, eight patients experienced masticatory muscle pain and fatigue within the first 10 days of therapy.

### Statistical analysis

A power analysis conducted using G*Power 3.1.9.2 (Franz Faul, Universität Kiel, Germany) determined that, to detect an effect size of 0.5 [25] with a Mann–Whitney test, a sample size of 52 subjects per group is required, assuming a significance level (α) of 0.05 and statistical power (1 − β) of 0.80.

Data distribution was assessed using the Shapiro-Wilk normality test. Descriptive statistics were calculated for all variables (Table 2). For within-group comparisons of polysomnographic indices and heart rate parameters before (T0) and after treatment (T1), the Wilcoxon signed-rank test was applied in cases of non-normal distribution, while the paired-sample t-test was used when data were normally distributed. Between-group differences in the T1 − T0 difference of each variable were analyzed using the Mann-Whitney U test.

To compare the effect of MAD and CPAP devices on heart rate (HR) variation, a propensity score matching (PSM) was performed. A 1:1 matching was conducted using Nearest Neighbor Matching based on two baseline pre-treatment variables: AHI and ODI. The propensity score was calculated through logistic regression, and subjects were matched to minimize the distance between propensity scores. Subsequently, pre- and post-treatment differences were analyzed for the following outcomes: ΔHRmin, ΔHRmean, and ΔHRmax.

**Fig. 2:**
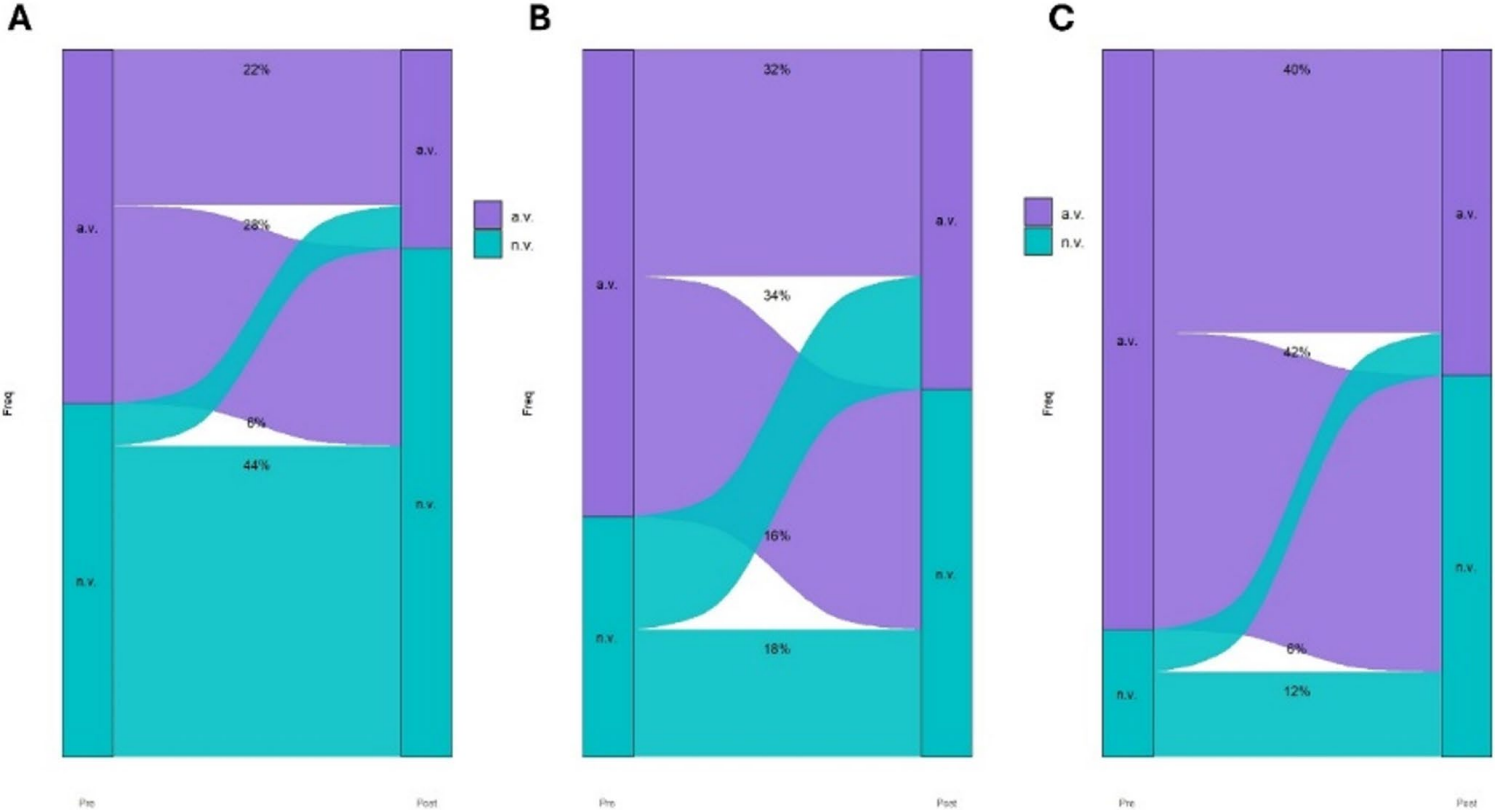
Sankey plot to show mean (**A**), minimum (**B**) and maximum (**C**) heart rate changes in CPAP-treated patients

**Fig. 3:**
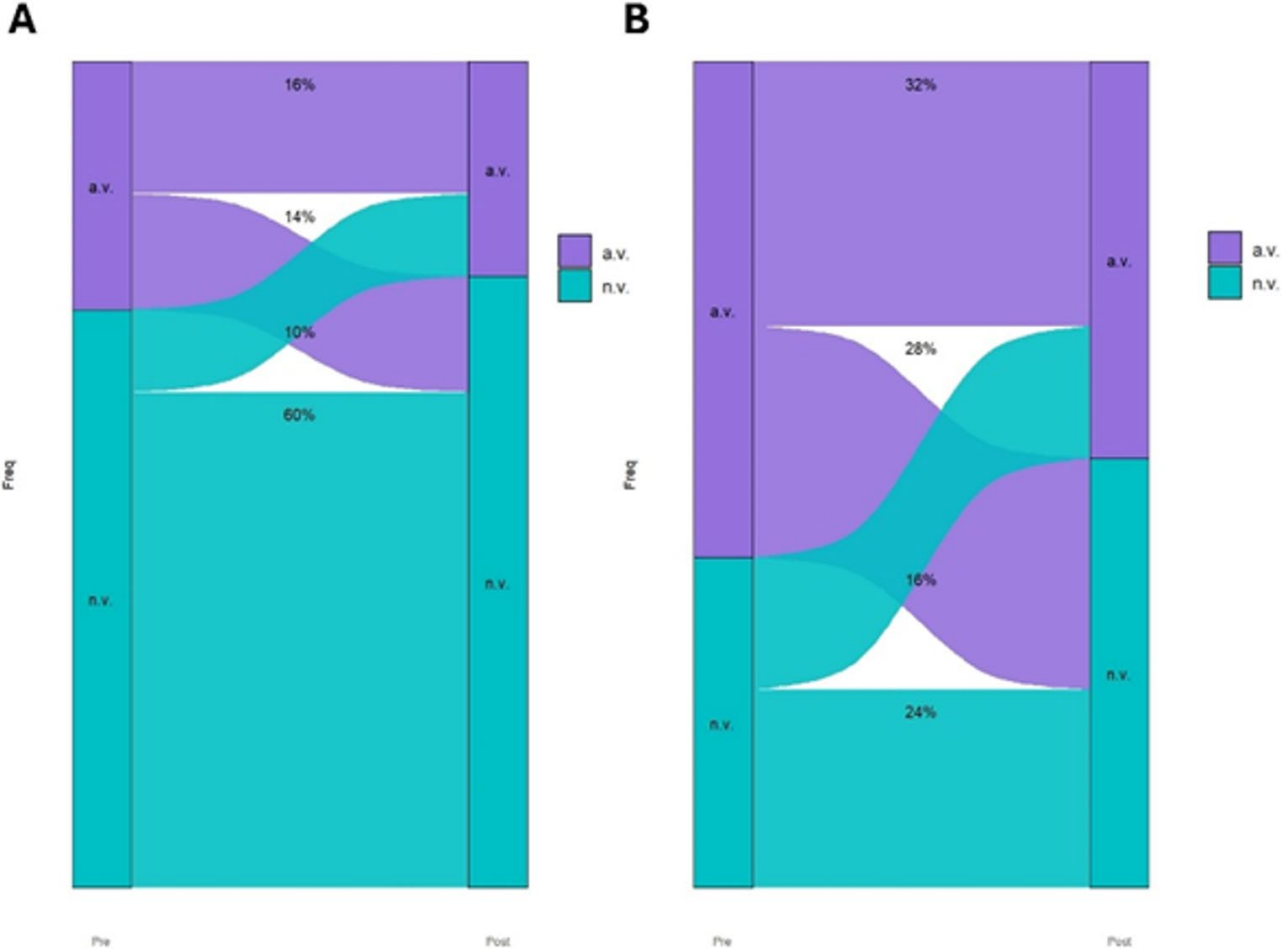
Sankey plot to show mean (**A**) and minimum (**B**) heart rate changes in MAD treated patients

Furthermore, in consideration of studies on the nocturnal circadian profile of cardiac function [26], Sankey diagram (Figs. 2 and 3) was constructed considering the following HR values as normal during sleep: HR mean 55–70 bpm, HR min 45–55 bpm and HR max 70–90 bpm. X2 test was used to compare the categorical variables.

All statistical analyses and graphs were performed using RStudio version 2025.05.1 + 513. Statistical significance was set at *p* < 0.05.

## Results

### Population

The patients treated with the MAD (MAD group) included 15 females and 37 males, with a mean age of 61.7 years. Baseline RPG showed AHI 27.12 ± 12.94 events/h, ODI 18.64 ± 9.01 events/h, HRmin 42.92 ± 10.55 bpm, HRmean 61.38 ± 7.40 bpm and HRmax 136.88 ± 34.30 bpm (Table 1).

**Table 1:** Descriptive statistics

	MAD			CPAP		
Variables	T0	T1	Δ(T1-T0)	T0	T1	Δ(T1-T0)
AHI	27.12 ± 12.94	7.03 ± 6.89	−20.09±−6,05	54.5 ± 22.93	6.74 ± 6.24	−47.76±−16.69
ODI	18.64 ± 9.01	5.14 ± 5.31	−13.5±−3.7	46.5 ± 26.05	7.22 ± 6.85	−39.28±−19.2
HRmean	61.38 ± 7.4	59.38 ± 7.2	−2±−0.2	69.83 ± 8.91	65.93 ± 7.59	−3.9±−1.32
HRmin	42.92 ± 10.55	43.82 ± 12.17	0.9 ± 1.62	46.36 ± 12.04	49 ± 13.45	2.64 ± 1.41
HRmax	136.88 ± 34.3	124.84 ± 37.83	−12.04 ± 3.53	104.38 ± 22.22	94.68 ± 23.97	−9.7 ± 1.75

Abbreviations: AHI = apnea-hyponea index, HR = heart rate, ODI = oxygen desaturation index

The patients treated with CPAP (CPAP group) comprised 14 females and 38 males, with a mean age of 61.5 years. AHI was 54.50 ± 22.93 events/h and ODI 46.50 ± 26.05 events/h. The sleep heart parameters were: HRmean 69.83 ± 8.91 bpm, HRmin 46,36 ± 12,04 bpm and HRmax 104,38 ± 22,22 bpm (Table 2).

### Intra-group comparison of treatment effects

Within-group statistical comparisons between baseline (T0) and post-treatment (T1) are shown in Table 2.

**Table 2:** Intra-Group comparison of treatment effects

	MAD			CPAP		
Variables	T0	T1	*P*	T0	T1	*P*
AHI	27.12 ± 12.94	7.03 ± 6.89	< 0.001	54.5 ± 22.93	6.74 ± 6.24	< 0.001
ODI	18.64 ± 9.01	5.14 ± 5.31	< 0.001	46.5 ± 26.05	7.22 ± 6.85	< 0.001
HRmean	61.38 ± 7.4	59.38 ± 7.2	0.033	69.83 ± 8.91	65.93 ± 7.59	0.002
HRmin	42.92 ± 10.55	43,82 ± 12.17	0.602	46.36 ± 12.04	49 ± 13.45	0.2
HRmax	136.88 ± 34.3	124,84 ± 37.83	0.049	104.38 ± 22.22	94.68 ± 23.97	0.034

In the MAD group, a statistically significant reduction was noted in the AHI, which decreased from 27.12 ± 12.94 to 7.03 ± 6.89 (*p* < 0.001), and in the ODI, which decreased from 18.64 ± 9.01 to 5.14 ± 5.31 (*p* < 0.001). A mild but statistically significant decrease was also detected in the mean HR (from 61.38 ± 7.40 to 59.38 ± 7.20 bpm; *p* = 0.033). No significant changes were found for HRmin (*p* = 0.602), whereas HRmax decreased significantly (from 136.88 ± 34.30 to 124.84 ± 37.83 bpm; *p* = 0.049).

Similarly, the C-PAP group showed a highly significant reduction in AHI (from 54.50 ± 22.93 to 6.74 ± 6.24; *p* < 0.001) and ODI (from 46.50 ± 26.05 to 7.22 ± 6.85; *p* < 0.001). The mean HR also showed a statistically significant decrease (from 69.83 ± 8.91 to 65.93 ± 7.59 bpm; *p* = 0.002). No significant change was found in HRmin (*p* = 0.2), while HRmax was significantly reduced (from 104.38 ± 22.22 to 94.68 ± 23.97 bpm; *p* = 0.034).

### Inter-group comparison of treatment outcomes

 As shown in Table 3, both treatment modalities resulted in a significant reduction in the AHI and ODI values. However, the baseline values of the AHI and the ODI were substantially higher in the CPAP group (AHI: 54.50 ± 22.93 events/h; ODI: 46.50 ± 26.05 events/h) compared to the MAD group (AHI: 27.12 ± 12.94 events/h; ODI: 18.64 ± 9.01 events/h), indicating a greater initial severity of OSA in the CPAP cohort.

**Table 3:** Inter-Group comparison of treatment outcomes

	MAD		CPAP		*P*
Variables	T0	T1	T0	T1	
AHI	27.12 ± 12.94	7.03 ± 6.89	54.50 ± 22.93	6.74 ± 6.24	< 0.001
ODI	18.64 ± 9.01	5.14 ± 5.31	46.50 ± 26.05	7.22 ± 6.85	< 0.001
HRmean	61.38 ± 7.40	59.38 ± 7.20	69.83 ± 8.91	65.93 ± 7.59	0.350
HRmin	42.92 ± 10.55	43.82 ± 12.17	46.35 ± 12.04	49 ± 13.45	0.602
HRmax	136.88 ± 34.30	124.84 ± 37.83	104.38 ± 22.22	94.68 ± 23.97	0.942

Regarding heart rate parameters, no statistically significant differences were found between the two groups in terms of mean HR (p = 0.350), HRmin (p = 0.602), or HRmax (p = 0.942) following treatment.

Subsequently, the comparison between the MAD and CPAP groups was performed after PSM. The analysis revealed a statistically significant difference in the variation of minimum HR (p < 0.001) and mean HR (p = 0.041), both in favor of the CPAP group. Conversely, no significant difference was found regarding maximum HR (p = 0.481).

 By considering the normality cutoffs of nocturnal HR as explained in the statistical analysis section, we observed that at baseline, patients subsequently treated with CPAP had altered values (a.v.) of mean, min and max HR in 50%, 66% and 82%, respectively (Fig. 2).

## Discussion

This study confirms that the effectiveness of both CPAP and MAD therapies in reducing respiratory events during sleep and improving gas exchange. It also suggests a potentially significant role for both treatments in modulating night-time heart rate fluctuations.

Notably, although CPAP therapy produced greater absolute improvements, likely reflecting the higher baseline severity in that group (mean AHI: 54.50 vs. 27.12 events/h), post-treatment values of AHI and ODI were comparable between groups, with no significant differences at follow-up (T1). These findings align with previous literature, which supports MAD as a viable alternative to CPAP, particularly in mild-to-moderate OSA, and even in selected cases of severe OSA [11]. Supporting this, a recent retrospective study by Ciavarella et al. [21] demonstrated that a fully customizable MAD significantly reduced AHI and ODI in adults with moderate-to-severe OSAS, alongside improvements in nadir and mean oxygen saturation levels (minSO₂ and medSO₂). This evidence underscores the potential of MAD to effectively mitigate respiratory disturbances across a broad severity spectrum. These findings further support the efficacy of the device, even in more severe cases of OSA.

Another relevant finding was the statistically significant reduction in mean and maximum HR observed in both groups after treatment, suggesting a beneficial effect on cardiovascular autonomic regulation during sleep. This improvement is likely related to the alleviation of intermittent hypoxia and sympathetic overactivity, both of which are induced by airway obstruction episodes in patients with OSA. Airway obstruction episodes in patients with OSA lead to the occurrence of apneas and hypopneas, as well as a reduction in blood oxygen levels. This negatively affects the autonomic nervous system, which regulates heart rate by balancing the actions of the sympathetic and parasympathetic systems. In patients with OSA, there is a chronic increase in sympathetic nervous system activation, resulting in elevated heart rate and blood pressure even during the daytime [27]. By chronically increasing sympathetic activation and reducing parasympathetic tone, resulting in elevated heart rate. These autonomic alterations are clinically relevant, as they contribute to increased cardiovascular risk and high mortality rate in OSA patients. A study by GrzędaHałon et al. investigated the impact of CPAP therapy on heart rate variability (HRV), a well-established non-invasive marker of autonomic nervous system function, in patients with OSA. The results showed that CPAP significantly modified several HRV parameters, with effects varying according to OSA severity, suggesting a shift in autonomic balance potentially linked to treatment-induced changes in airway obstruction severity [28]. Complementing this, Guo et al. (2018) conducted a meta-analysis revealing that CPAP treatment decreases both low-frequency (LF) and high-frequency (HF) HRV components, indicating enhanced sympatho-vagal balance during sleep [29]. Moreover, Pamidi et al. (2020) found that nocturnal CPAP therapy reduces daytime resting heart rate in adults with prediabetes, suggesting that CPAP’s autonomic benefits extend beyond sleep into wakefulness [30, 31]. The results of our study are consistent with these evidences, reinforcing the hypothesis that CPAP therapy positively modulates autonomic function and contributes to cardiovascular protection in this patient population [32].

In our study, treatment with the MAD also resulted in a significant reduction in both mean HR (from 61.38 ± 7.40 to 59.38 ± 7.20 bpm; *p* = 0.033) and maximum HR (from 136.88 ± 34.30 to 124.84 ± 37.83 bpm; *p* = 0.049). These findings are consistent with the study by Ciavarella et al., which reported a reduction in maximum HR following MAD therapy [33], as well as with the study by Glos et al., who found that CPAP and MAD therapies yield comparable outcomes in improving both respiratory parameters and autonomic regulation [34].

This study confirms that that both MAD and CPAP significantly improve HR in patients with OSA, reflecting decreased sympathetic nervous system activation. While CPAP acts by providing continuous positive airway pressure to keep the upper airway open throughout the night, MAD works mechanically by advancing the mandible to increase airway patency. However, despite their shared goal, CPAP demonstrated greater efficacy than MAD in improving HR, as confirmed by statistically significant differences observed.

### Limitations of the study

This study has several limitations that should be acknowledged. Indeed, the small sample size, which may limit the generalizability of the results. Furthermore, within the two treatment samples, baseline OSA severity differed. The CPAP group had higher initial AHI values, which could have influenced the magnitude of response to treatment. Given the retrospective design of this study, it is challenging to determine which unexamined variables may have impacted the results. Future research with larger cohorts, standardized adherence monitoring, and longer follow-up periods is needed to confirm and extend these findings.

## Conclusion

The use of both MAD and CPAP therapies leads to improvements in heart rate regulation in patients with OSA, suggesting beneficial effects on autonomic cardiovascular control. However, CPAP demonstrated a greater capacity to improve heart rate compared to MAD.

These findings underscore the importance of a personalized treatment strategy in OSA, one that considers not only respiratory outcomes but also cardiovascular impact, baseline severity, and patient adherence, to optimize clinical management and reduce long-term cardiovascular risk.

## Supplementary Information

Below is the link to the electronic supplementary material.

Supplementary Material 1(DOCX 83.5 KB) 

## Data Availability

The data that support the findings of this study are available from the corresponding author upon reasonable request.
